# Effect of GABA-T on Reproductive Function in Female Rats

**DOI:** 10.3390/ani10040567

**Published:** 2020-03-27

**Authors:** Wenyu Si, Hailing Li, Tiezhu Kang, Jing Ye, Zhiqiu Yao, Ya Liu, Tong Yu, Yunhai Zhang, Yinghui Ling, Hongguo Cao, Juhua Wang, Yunsheng Li, Fugui Fang

**Affiliations:** 1 Anhui Provincial Laboratory of Animal Genetic Resources Protection and Breeding, College of Animal Science and Technology, Anhui Agricultural University, 130 Changjiang West Road, Hefei 230036, China; wenyusi0411@163.com (W.S.); lhlingwww@163.com (H.L.); ktz1021@163.com (T.K.); yejing350698@163.com (J.Y.); zhiqiuyao1@163.com (Z.Y.); liuya@ahau.edu.cn (Y.L.); yt8504@gmail.com (T.Y.); yunhaizhang@ahau.edu.cn (Y.Z.); lingyinghui@ahau.edu.cn (Y.L.); caohongguo1@126.com (H.C.); wjhxxh@163.com (J.W.); lys@ahau.edu.cn (Y.L.); 2 Anhui Provincial Laboratory for Local Livestock and Poultry Genetic Resource Conservation and Bio-Breeding, 130 Changjiang West Road, Hefei 230036, China; 3 Department of Animal Veterinary Science, College of Animal Science and Technology, Anhui Agricultural University, 130 Changjiang West Road, Hefei 230036, China

**Keywords:** GABA-T, hypothalamus-pituitary-ovarian axis (HPOA), puberty, reproductive performance, reproductive hormones, rat

## Abstract

**Simple Summary:**

This study evaluated the effect of γ-aminobutyric acid transaminase (GABA-T) on reproduction in female rats. Our results showed that GABA-T expressed in the reproductive axis of female rats at different developmental stages, and inhibiting GABA-T affected *Rfrp-3*, *Gnrh* and *Kiss1* mRNA transcription levels in hypothalamic and reduced the concentration of GABA-T, LH, and P4 in serum. At the same time, the initiation of the puberty was delayed, and led to disordered estrous cycle and reduced breastfeeding performance in adult female rats. This study showed that GABA-T affects the reproductive function of female rats by regulating the levels of *Gnrh*, *Kiss1* and *Rfrp-3* in the hypothalamus as well as the concentrations of LH and P_4_.

**Abstract:**

This study explored the role of γ-aminobutyric acid transaminase (GABA-T) in the puberty and reproductive performance of female rats. Immunofluorescence technique, quantitative real-time PCR (RT-qPCR) and enzyme-linked immunosorbent assay (ELISA) were used to detect the distribution of GABA-T and the expression of genes and hormones in female rats, respectively. The results showed that GABA-T was mainly distributed in the arcuate nucleus (ARC), paraventricular nucleus (PVN) and periventricular nucleus (PeN) of the hypothalamus, and in the adenohypophysis, ovarian granulosa cells and oocytes. *Abat* mRNA level at 28 d was lowest in the hypothalamus and the pituitary; at puberty, it was lowest in the ovary. *Abat* mRNA level was highest in adults in the hypothalamus; at infancy and puberty, it was highest in the pituitary; and at 21 d it was highest in the ovary. After vigabatrin (GABA-T irreversible inhibitor) was added to hypothalamus cells, the levels of *Abat* mRNA and *Rfrp-3* mRNA were significantly reduced, but *Gnrh* mRNA increased at the dose of 25 and 50 μg/mL; *Kiss1* mRNA was significantly increased but *Gabbr1* mRNA was reduced at the 50 μg/mL dose. In prepubertal rats injected with vigabatrin, puberty onset was delayed. *Abat* mRNA, *Kiss1* mRNA and *Gnrh* mRNA levels were significantly reduced, but *Rfrp-3* mRNA level increased in the hypothalamus. Vigabatrin reduced the concentrations of GABA-T, luteinizing hormone (LH) and progesterone (P_4_), and the ovarian index. Lactation performance was reduced in adult rats with vigabatrin treatment. Four hours after vigabatrin injection, the concentrations of GABA-T and LH were significantly reduced in adult and 25 d rats, but follicle-stimulating hormone (FSH) increased in 25 d rats. In conclusion, GABA-T affects the reproductive function of female rats by regulating the levels of *Gnrh*, *Kiss1* and *Rfrp-3* in the hypothalamus as well as the concentrations of LH and P_4_.

## 1. Introduction

Reproduction is the process by which an animal breeds and produces offspring. Puberty is essential in the growth and development of animals, and is an important symbol of reproductive ability [1]. After the onset of puberty, the reproductive organs become mature and begin to perform reproductive activities. The onset of puberty is regulated by the hypothalamic–pituitary–gonadal axis (HPGA) [2]. The increase in gonadotropin-releasing hormone (GnRH) released in the hypothalamus before puberty results in the increased release of luteinizing hormone (LH) and follicle-stimulating hormone (FSH) in the anterior pituitary; both hormones act on the gonad, stimulating their development, gametogenesis and sexual steroid production [3]. Many factors can affect animal puberty and reproduction. The system of kisspeptin-G protein-coupled receptor 54 (kiss1-GPR54) [4], γ-aminobutyric acid (GABA) [5], leptin [6], D-Aspartate [7] and others are essential in regulating reproduction. The receptor GPR147 of RFRP-3 is expressed in the subsets of GnRH and kisspeptin neurons, which can inhibit kisspeptin neurons and GnRH neurons by the central mechanism to reduce estrus in rats and hamsters [8,9]. Kisspeptin is the main regulator of GnRH and is essential in initiation, sex hormone secretion and growth and development during puberty [10]. Research has shown that the HPGA is regulated by GABAergic signaling at the level of GnRH neurons [11], and GnRH is an important regulatory hormone in animal reproductive development.

GABA is a major amino acid in vertebrates. In 1950, Roberts and Frankel discovered that GABA is present in nerve tissue and functions directly in the regulation of neural centers [12]. GABA functions keep the brain strong, and it has anti-anxiety, analgesic, hypnotic and forgetting effects [13,14]. Recent studies have shown that GABA inhibits the secration of luteinizing hormone (LH) and prolactin (PRL) in the anterior pituitary gland [15], and GABA has been found in more than 30 peripheral tissues, including ovarian and fallopian tubes [16]. Other studies have shown that GABA receptors (GABAA and GABAB) can stop the sperm acrosome reaction [17] and GABA regulates follicular development and pregnancy [16]. GABA inhibits the onset of puberty and reproduction of mice [18] and monkeys [19] by regulating the secretion of GnRH. All these results indicate that GABA is involved in reproductive function. 

γ-aminobutyric acid transaminase (GABA-T) is formed by the *Abat* gene, which can decompose GABA into glutamic acid and succinic semialdehyde, and then convert it into succinic acid to enter the tricarboxylic acid cycle for metabolism [20]. The anticonvulsant γ-vinyl GABA (vigabatrin) is an irreversible antagonist of GABA-T that induces an increase in GABA levels in rat, mice and human brains [21,22,23,24]. These results all indicate that GABA-T functions directly in GABA metabolism in the central nervous system. GABA is known to regulate reproduction, but it is not known whether GABA-T affects reproductive function by affecting GABA. GABAergic signaling pathways exhibited an association with the initiation of puberty, and GABA-T was significantly increased in pubertal goats compared to prepubertal goats [25,26]. Therefore, we speculated that GABA-T affects reproductive function. 

However, research on GABA-T has been focused on mental illness, such as epilepsy [20]. No data are available regarding the role of GABA-T in the onset of puberty in animals or in reproductive performance. Thus, the present study was aimed at gaining further insights into the role of GABA-T in the reproductive function of female animals. Using female rats as experimental animals, we focused on five specific objectives: (1) How does the distribution and expression of GABA-T change in the HPGA during different developmental stages of rats? (2) What are the roles of GABA-T in the regulation of GnRH and reproduction-related genes in hypothalamic neurons in vitro? (3) What are the functions of GABA-T in mediating reproductive hormones and genes of the gonadal axis in pubertal rats in vivo? (4) How does GABA-T regulate reproductive performance in adult female rats? (5) What are the effects of GABA-T on the hormones of reproduction-related genes in rats over a short time? The results of the present study will help us better understand the role of GABA-T in reproduction.

## 2. Materials and Methods

### 2.1. Animals

Healthy adult Sprague-Dawley rats were bred at the animal center of Anhui Agricultural University. The rats were housed under a controlled temperature (20–25 °C) and with continuous provision of a light/dark cycle of 12/12 h; water and food were provided freely. Adult rats were kept according to a ratio of 1:1 male (70 d) to female (65 d), and after 10 days female rats were raised separately and were ready for reproduction. The day of litter birth was considered age day 1 (postnatal day 1, PND1), and rats were weaned at PND21. All experimental procedures involving rats were performed according to the Regulations for the Administration of Affairs Concerning Experimental Animals (Ministry of Science and Technology, China; revised in June 2004).

### 2.2. Experimental Design

#### 2.2.1. Distribution of GABA-T protein and *Abat* mRNA Level in The HPG Axis at Different Developmental Stages

The hypothalamus, pituitary and ovary were collected from female rats at infant (10 d), prepubertal (21 d and 28 d), pubertal (the first day of vaginal opening (VO) 33.67 ± 0.49 d) and adult (more than 60 d and in diestrus, 64.16 ± 0.79 d) stages [27]. Collected samples were stored in liquid nitrogen for the extraction of RNA. Tissue samples were fixed in 4% paraformaldehyde for 4 h and stored at 4 °C until used for immunofluorescence.

#### 2.2.2. Effect of GABA-T inhibitor on The Expression of Reproduction-related Genes in Hypothalamus Cells

Eighteen female rats at PND1 were sterilized with alcohol and then anesthetized with sodium pentobarbital [28,29]. Then, heads were separated and placed in a 10 cm petri dish, the dish was subsequently brought into the cell room and put in a clean bench. The brain was taken out in an ice box in a 6 cm dish containing 4 mL Dulbecco’s modified Eagle’s medium (DMEM) (Hyclone, USA); afterward, the hypothalamus was separated from the brain and kept in a 1.5 mL centrifuge tube with DMEM (three hypothalami per tube). The hypothalamus was cut into pieces with an ophthalmic scissor and allowed to stand for 2 min, after which the supernatant was discarded. Trypsin (0.125%) (Gibco, USA) was added to digest for 15–20 min, and the supernatant was discarded after centrifugation. Two mL of DMEM with 20% fetal bovine serum (FBS) (Sijiqing, China) was mixed in; later, the mixture was filtered through a 200-mesh filter and up to 36 mL was collected. Then, the mixture was added into three 6-well plates to culture (2 mL per well). On the next day, the culture medium was changed to Neurobasal™ medium (Gibco, USA) containing 2% B-27 (Gibco, USA), and the liquid was changed every other day until the cell plating area was about 75% per well. The GABA-T inhibitor (vigabatrin) (HY-15399, MedChem Express, USA) was dissolved in ddH_2_O, then added to the cultures, which were cultured for 20–24 h, followed by RNA extraction. Samples were divided into 25 and 50 μg/mL inhibitor groups and a ddH_2_O control group.

#### 2.2.3. Effect of GABA-T inhibitor on Puberty Onset, Reproduction-related Genes, Hormones and Ovarian Morphology in Female Rat

Twelve PND25 rats were randomly and equally divided into two groups (inhibitor group (1 μg/g) and control group) for lateral ventricle injection. Rats were observed daily for VO, and VO time was recorded. On the day of VO, all rats were anesthetized with sodium pentobarbital [30] and then sacrificed to collect the samples. Serum was separated for enzyme-linked immunosorbent assay (ELISA). The hypothalamus, pituitary and ovaries were collected and stored at −80 °C for real-time PCR (RT-qPCR). Ovaries were weighed and fixed in 4% paraformaldehyde for histological sectioning. 

#### 2.2.4. Effect of GABA-T inhibitor on Estrous Cycle and Reproductive Performance in Adult Female Rats

Twenty adult rats during diestrus (64.46 ± 0.46 d) were divided into two groups. The inhibitor group was intracerebroventricularly injected in the hypothalamus with vigabatrin at a dose of 1 μg/g, and the control group was injected with an equal volume of ddH_2_O. The estrous cycle of the rats was observed by vaginal smears taken from 9:00 to 11:00 a.m. every day for 18 consecutive days. Adult male (85 d) and female rats (82.46 ± 0.46 d) were then mated according to a 1:1 ratio, and the breeding time was 21 days (adult male rats can produce offspring in previous matings, indicating that they have no reproductive disorders). The mating time of the rats (through vaginal smears, observation of the presence or absence of sperm), pregnancy time, and numbers and weight of the offspring were determined.

#### 2.2.5. The Short-term Effect of GABA-T inhibitor for 4 h on Reproduction-related Genes and Hormones in Rats

Twelve PND25 rats and twelve adult rats during diestrus (64.58 ± 0.52 d) were divided into an inhibitor group (1 μg/g) and control group. After 4 h of vigabatrin injection, blood was collected from all rats for ELISA, and the hypothalamus, pituitary and ovary were collected for RT-qPCR.

### 2.3. Lateral Ventricle Injection

All experimental instruments and countertops were disinfected, and female rats were fixed in the stereotaxic apparatus after they were anesthetized with 2% sodium pentobarbital (0.02 mL/10 g body weight) [31]. Hair was removed from the head and the head was disinfected with ethanol, then the skin and periosteum were dissected to expose the bregma point, and a microsyringe was inserted into the skull at a 90° angle (parameters: 2.0–2.5 mm rostral from bregma, 0.5–1.0 mm lateral from midline, and 8.6–9.0 mm ventral from the surface of the skull) [32]. The syringe was kept in place for 5 min and then slowly injected at a speed of 0.15 µl/min. The syringe was retained for another 5 min before it was removed, the wound was sutured, and an injection of penicillin was given (10,000 units each rat).

### 2.4. Vaginal Smear and Wright’s Giemsa Staining

A cotton swab moistened with physiological saline was gently placed in the vagina of the rat, rotated, and then removed, and the swab was spread evenly on a glass slide. Giemsa dye was applied on the glass slide for about 1 min, then PBS was added dropwise, and the slide was left standing for 5 min. Finally, the slide was rinsed with water, dried and observed under a microscope.

### 2.5. Serum Preparation and ELISA

Blood was collected from female rats, and the serum was separated by centrifugation at 3000 rpm for 15 min at 4 °C, and stored at −20 °C. The contents of GABA-T, GABA, estradiol (E_2_), progesterone (P_4_), LH and FSH were determined according to the kit instructions for the ELISA. A standard solution of 50 μL and serum samples was added to each well of a 96-well plate, excluding two blank wells (no added enzyme-labeled reagents and samples), then 100 μL of an enzyme labeling reagent was added and samples were mixed gently and then incubated at 37 °C for 60 min. The solution was then removed and wells were washed five times, then 100 μL of substrate reagent was added and the plates were incubated for 15 min at 37 °C in the dark. The reaction was stopped by adding 50 μL of the stop solution, and the absorbance (OD) was determined at 450 nm immediately using a Pan-wavelength Multimode Reader (Elabscience Biotechnology Co., Ltd., Wuhan, China) [33]. The sensitivity of GABA-T (KS18477, KESHUN, China) and P_4_ (KS18474, KESHUN, China) were 0.1 ng/mL; GABA (KS12343, KESHUN, China) was 0.1 μmol/L; E_2_ (KS11525, KESHUN, China) was 0.1 pmol/L; and LH (KS18265, KESHUN, China) and FSH (KS13251, KESHUN, China) were both 0.1 IU/L. The intra-assay and inter-assay coefficient of variation of all hormones was less than 10% and 15%, respectively; the coefficient of the standard curves was above 0.9900.

### 2.6. Immunofluorescence Staining

The hypothalamus, pituitary and ovary of rats at different stages fixed in 4% paraformaldehyde were taken out, dehydrated with different concentrations of alcohol, cleared by xylene, and embedded in paraffin. The sections were cut into a thickness of 5 μm, and immersed in alcohol and xylene to remove paraffin, and citrate to repair antigen. Then, 10% bovine serum albumin (BSA), (G5001, Servicebio, China) was added dropwise and incubated at room temperature for 30 min to block non-specific antigens. The sections were incubated with rabbit anti-GABA-T antibody (1:100 dilution; EPR4433, Abcam, Britain) overnight at 4 °C. After incubation, the slides were heated in a 37 °C incubator for 40 minutes and reacted with donkey anti-shine immunoglobulin (Ig) G-fluorochrome NL557 (1:200 dilution; NL010, R&D Systems, USA) secondary antibodies [34]. Sections were then incubated at 37 °C for 1 hour at room temperature in the dark. The cells were immersed in PBS three times and Vectashield mounting media containing 4,6-diamidino-2-phenylindole (DAPI; blue nuclear dye; Vector Laboratories) was added [35]. The negative controls were not incubated with a primary antibody and were only treated with a secondary antibody.

### 2.7. Hematoxylin and Eosin (H&E) Staining

The ovaries fixed in 4% paraformaldehyde were taken out, dehydrated with different concentrations of alcohol, cleared by xylene, and embedded in paraffin. The paraffin-embedded tissue sections were cut into 5 μm thickness, dewaxed, dehydrated and stained with H&E. 

### 2.8. RNA Extractions and Reverse Transcription

Total RNA was extracted from the hypothalamus, pituitary and ovary using the Total RNA Kit II R6934 (OMEGA, Guangzhou, China); and cell RNA was extracted using the Total RNA Kit 6831 (OMEGA, Guangzhou, China) from hypothalamus cells. Then, RNA quality was examined using a Nano Drop ND-2000 Spectrophotometer (Nano-Drop, USA), and agarose gel electrophoresis. After that the RNA was concentrated and 500 ng of each sample was purified and reverse-transcribed into cDNA using EasyScript One-Step gDNA Removal and cDNA Synthesis SuperMix (Trans, Beijing, China). qPCR was performed in a final mixture of 20 µL.

### 2.9. RT-qPCR

The PCR conditions were as follows: initial denaturation at 95 °C for 1 min, 40 cycles of denaturation at 95 °C for 10 s, annealing at 60 °C for 30 s, and extension at 72 °C for 1 min. A final extension step was included at 72°C for 7 min. Each qPCR reaction was performed in triplicate. The primers were synthesized by Shanghai Sangon Biotech. *GAPDH* and *β-actin* were used as internal controls (Table 1). The 2^–ΔΔCT^ method was used to calculate the relative expression levels of genes [36].

### 2.10. Statistical Analysis

The Statistical Package for the Social Sciences (SPSS) version 21.0 was used for analysis. Results were expressed as the means ± standard error of the mean (SEM) Differences between groups were compared by one-way ANOVA, *p*-values < 0.05 were considered to indicate statistical significance.

## 3. Results

### 3.1. Level of Abat mRNA and Distribution of GABA-T Protein on The Reproductive Axis of Female Rats

**Figure 1:**
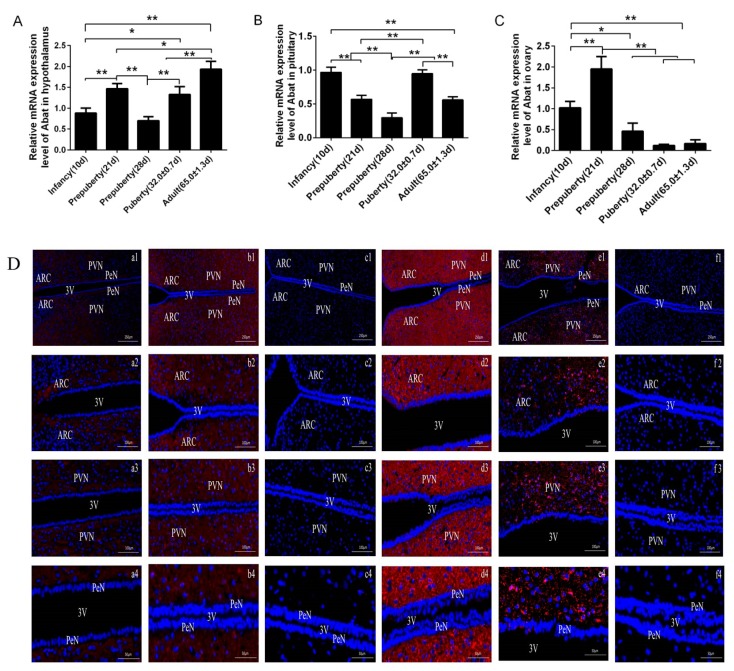

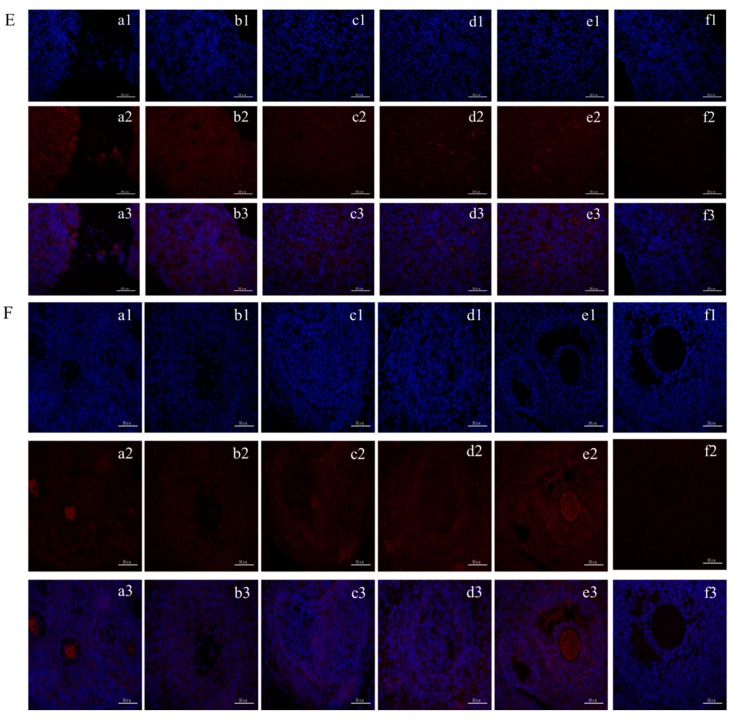
Level of *Abat* mRNA and distribution of GABA-T protein in hypothalamus, pituitary and ovary of female rats from infancy to adult. (**A**) Relative *Abat* mRNA expression levels in the hypothalamus from infancy to adult. (**B**) Relative *Abat* mRNA expression levels in the pituitary from infancy to adult. (**C**) Relative *Abat* mRNA expression levels in the ovary from infancy to adult. ** indicates a very significant difference (*P* < 0.01, n = 6); * indicates a significant difference (*P* < 0.05, n = 6). (**D**) Distribution of GABA-T in hypothalamus. Arcuate nucleus: ARC; Paraventricular nucleus: PVN; Periventricular nucleus: PeN; Third ventricle: 3V; a1-4: Infancy(10d); b1-4: Prepuberty (21d); c1-4: Prepuberty (28d); d1-4: Puberty (33.67 ± 0.49 d); e1-4: Adult (64.16 ± 0.79 d); f1-4: Negative control; The scale bar of 1, 2, 3, 4 are 250 μm, 100 μm, 100 μm, 50 μm. (**E**) and (**F**) Distribution of GABA-T in the pituitary and ovary. 1: DAPI; 2: GABA-T immunofluorescence staining (red fluorescence); 3: Merge; a1-3: Infancy(10d); b1-3: Prepuberty (21d); c1-3: Prepuberty (28d); d1-3: Puberty (33.67 ± 0.49 d); e1-3: Adult (64.16 ± 0.79 d); f1-3: Negative control; Scale bar: 50 μm.

The expression of *Abat* mRNA at different developmental stages of rats in the hypothalamus, pituitary and ovary is shown in Figure 1. In the hypothalamus (Figure 1A), *Abat* mRNA level was significantly reduced at infancy, 28 d, puberty (*p* < 0.01) and 21 d (*p* < 0.05) rats compared with adults; the level of *Abat* mRNA at infancy and 28 d were significantly lower than that at 21 d and puberty (*p* < 0.01). In the pituitary (Figure 1B), the level of *Abat* mRNA was the lowest at 28 d, which was significantly lower than that at infancy, 21 d, puberty and adult (*p* < 0.01); there was no significant difference in the expression of *Abat* mRNA between infancy and puberty (*p* > 0.05), but, compared with these two periods, the expression of *Abat* mRNA was significantly reduced at 21 d and adult stages (*p* < 0.01). In the ovary (Figure 1C), compared with 21 d, the *Abat* mRNA level were significantly reduced in infancy, 28 d, puberty and adult (*p* < 0.01); the *Abat* mRNA level in infancy was significantly higher than that at 28 d (*p* < 0.05), puberty and adult (*p* < 0.01). No differences were found between 28 d, puberty and adult (*p* > 0.05). 

Results showed that GABA-T was expressed in the arcuate nucleus (ARC), paraventricular nucleus (PVN) and periventricular nucleus (PeN) of the rat hypothalamus (Figure 1D) at different developmental stages. In the pubertal rat, the immunofluorescence intensity of GABA-T was the strongest, showing a dense, patchy distribution; the immunofluorescence intensity at 21 d and adult was second strongest; and the 28 d immunofluorescence was the lowest, with almost no red fluorescence. As shown in Figure 1, GABA-T was distributed on the adenohypophysis (Figure 1E) and ovarian granulosa cells and oocytes (Figure 1F) at different developmental stages. There was no positive fluorescence reaction in the negative control group (Figure 1D–F). 

### 3.2. Effects of GABA-T inhibitor on Reproduction-related Genes in Rat Hypothalamic Cells

**Figure 2:**
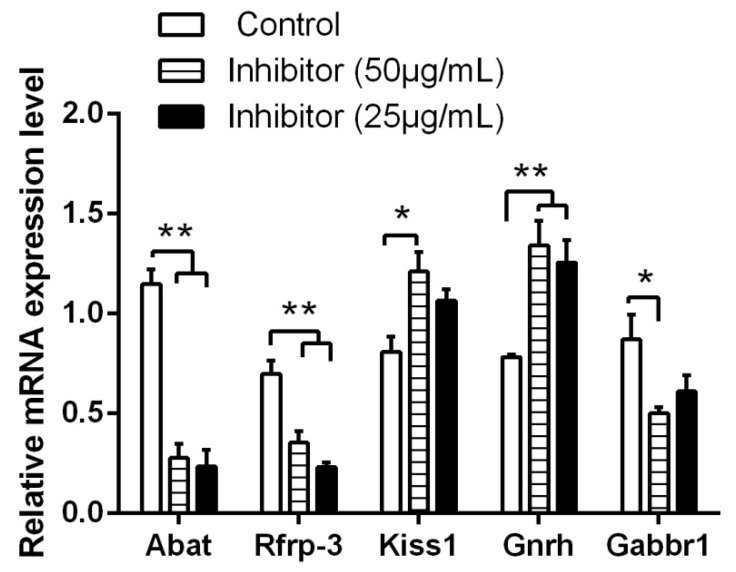
Effects of GABA-T inhibitors on the expression of reproduction-related genes in hypothalamic neurons. ** indicates a very significant difference (*p* < 0.01); * indicates a significant difference (*p* < 0.05). Vigabatrin was used as a GABA-T inhibitor.

The addition of vigabatrin to hypothalamic cells for 24 h showed that, at the doses of 50 and 25 μg/mL vigabatrin, the expressions of *Abat* and *Rfrp-3* mRNA were reduced (*p* < 0.01) and *Gnrh* mRNA increased (*p* < 0.01) compared to those in the control group (*p* < 0.01) (Figure 2). We also found that *Kiss1* mRNA level was significantly increased (*p* < 0.05) and *Gabbr1* mRNA level decreased (*p* < 0.05) at the dose of 50 μg/mL vigabatrin. The expressions of *Kiss1* and *Gabbr1* mRNA at the 25 μg/mL dose were not different from the control group (*p* > 0.05). No significant differences were found between 50 and 25 μg/mL doses in the expression of *Abat*, *Rfrp-3*, *Kiss1*, *Gnrh* and *Gabbr1* mRNA (*p* > 0.05).

### 3.3. Effects of GABA-T Inhibitor on Puberty in Rats

#### 3.3.1. Changes in Time of Vaginal Opening

When the genital gland of the rat closes, this indicates that the animal is in the prepuberty stage; the puberty phase of the rat was characterized by moist vaginal opening (VO), inward depression, and open cavity (Supplementary Figure S1). Observing the vagina of the rats and counting the opening time of the vulva, it was found that the VO time in the inhibitor group was 35.30 ± 0.42d, which was significantly delayed by about three days compared with the VO time of the control group (32.40 ± 0.49d, *p* < 0.05). However, no behavioral abnormalities were observed in 25 d rats after vigabatrin injection.

#### 3.3.2. The Expression of Reproduction-related Genes

**Figure 3:**
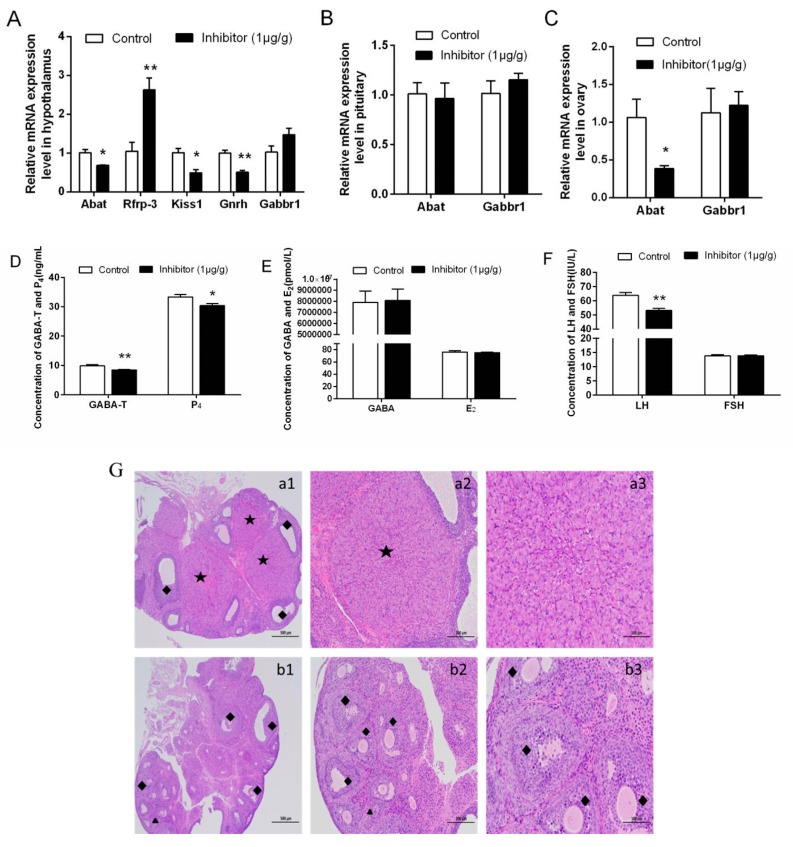
The reproduction-related genes expression, serum hormone content and ovarian morphology in 25d rats injected with GABA-T inhibitors until VO. (**A**) Expression of *Abat* mRNA and reproduction-related genes in hypothalamus at VO. (**B**) Expression of *Abat* and *Gabbr1* mRNA in pituitary at VO. (**C**) Expression of *Abat* and *Gabbr1* mRNA in ovary at VO. (**D**) The content of GABA-T and P_4_ in rat serum at VO. (**E**) The content of GABA and E_2_ in rat serum at VO. (**F**) The content of LH and FSH in rat serum at VO. (**G**) Histological assessment of follicles using H&E staining (a: Control group; b:GABA-T inhibitor group; the scale bar: of 1, 2, 3 are 500 μm, 200 μm, 100 μm). ** indicates a very significant difference (*p* < 0.01); * indicates a significant difference (*p* < 0.05). Vigabatrin was used as a GABA-T inhibitor.

Results in the hypothalamus (Figure 3A) showed significantly reduced *Abat* (*p* < 0.05)*, Kiss1* (*p* < 0.05) and *Gnrh* (*p* < 0.05) mRNA levels but *Rfrp-3* mRNA expression increased (*p* < 0.01) in the inhibitor group; the level of *Gabbr1* mRNA was not significantly different (*p* > 0.05) in the inhibitor group. In the pituitary (Figure 3B), there were no significant differences between control and inhibitor groups in *Abat* or *Gabbr1* mRNA levels (*p* > 0.05). In the ovary (Figure 3C), we found that *Abat* mRNA expression in the inhibitor group was significantly lower than that in the control group (*p* < 0.01), while there was no difference in *Gabbr1* mRNA expression between groups (*p* > 0.05). 

#### 3.3.3. Concentrations of Serum Hormones

Serum hormone concentrations of rats on the day of VO were detected by ELISA. GABA-T concentration (Figure 3D) and LH concentration (Figure 3F) were significantly lower in the inhibitor group than the control group (*p* < 0.01). P_4_ concentration (Figure 3D) was reduced significantly in the inhibitor group (*p* < 0.05). There were no significant differences in FSH (Figure 3F), GABA (Figure 3E) or E_2_ concentrations (Figure 3E) between groups (*p* > 0.05). 

#### 3.3.4. The Structure of Ovarian Histology

Rats at 25 d were injected with vigabatrin until VO, and we found that both the corpus luteum and the secondary follicles were present in the inhibitor group and the control group, but there were more secondary follicles in the inhibitor group, and the control group had more and larger corpus luteum (Figure 3G). The weight, transverse diameter, longitudinal diameter, transverse perimeter and longitudinal perimeter of ovaries were not significantly different between control and inhibitor groups (*p* > 0.05), but the ovary index of the inhibitor group was significantly lower than those of the control group (*p* < 0.05) (Table 2).

### 3.4. Effects of GABA-T Inhibitors on Estrous Cycle and Reproductive Performance in Adult Rats

#### 3.4.1. Changes in The Estrous Cycle

**Figure 4:**
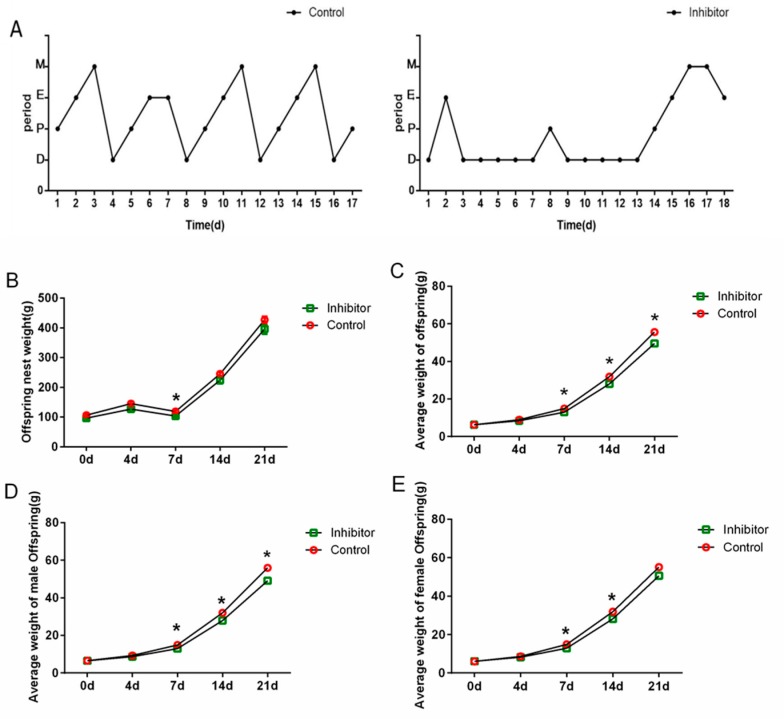
Effects of GABA-T inhibitor treatment on estrous cycle and offspring weight in adult female rats. M:metaoestrus, E:estrus, P:preoestrus, D:diestrus. (**A**) Changes in estrus cycle in adult female rats. The estrous cycle of the control group was normal, but the estrous cycle of the inhibitor group was disordered. (**B**) Offspring nest weight. (**C**) Offspring average weight. (**D**) Male offspring average weight. (**E**) Female offspring average weight. Adult rat age is 64.46 ± 0.46 d. Vigabatrin was used as a GABA-T inhibitor.

After injecting vigabatrin into the lateral ventricle, adult rats showed lethargy; they staggered and wobbled after waking, drinking and eating occasionally. This state lasted for 3–4 days. The degree of keratinization of vaginal cells was different over the estrous cycle in rats [27]. The diestrus period (Supplementary Figure S2DI) was characterized by the presence of a large number of neutrophils; the proestrus period (Supplementary Figure S2P) exhibited a large number of nucleated epithelial cells; the estrus period (Supplementary Figure S2E) showed nuclear epithelial cells transformed into keratinized flaky epithelial cells; the metestrus period (Supplementary Figure S2M) exhibited the simultaneous presence of three kinds of cells. The estrous cycle of rats was counted as shown in Figure 4A: the estrous cycle of the control group was normal, about 4 days; the estrous cycle of the inhibitor group was disordered, and the estrous cycle gradually returned to normal on the 15th during the 18-day observation period.

#### 3.4.2. Mating Time and Rate of Pregnancy

After the injection of vigabatrin into the hypothalamus, the successful mating time of the rats was determined (Table 3); all rats were mated in one estrous cycle. The first day of successful mating in rats was recorded as the first day of pregnancy, and the interval from the 1st day of gestation until the birth of the pups was considered as pregnancy. The gestation period of the control and inhibitor groups was 22–23 d (Table 4).

#### 3.4.3. Number and Growth of The Offspring

The number of litters after the injection of vigabatrin into the hypothalamus is shown in Table 5. There was no significant difference in litter size between the control group and the inhibitor group at different ages (*p* > 0.05).

Rat offspring were weighed to calculate nest weight and average body weight. At day 7, offspring nest weight in the inhibitor group was significantly lower than in the control group (*p* < 0.05); the differences on other days were not significant (*p* > 0.05) (Figure 4B). The average body weight of the offspring (Figure 4C) and the average body weight of the male offspring (Figure 4D) showed that the weights of the inhibitor group were reduced at 7, 14 and 21 d compared with the control group (*p* < 0.05); 0 and 4 d had no significant differences between the two groups (*p* > 0.05). Comparing the inhibitor group with the control group, the average weight of female offspring (Figure 4E) at 7 and 14 d were decreased (*p* < 0.05); no differences were found between the two groups at 0, 4 or 21 d (*p* > 0.05).

### 3.5. The Short-term Effects of GABA-T Inhibitor on Reproduction-related Genes and Hormones

#### 3.5.1. Expression of Reproduction-related Genes in The Reproductive Axis After 4 h of GABT-T Inhibitor Treatment

**Figure 5:**
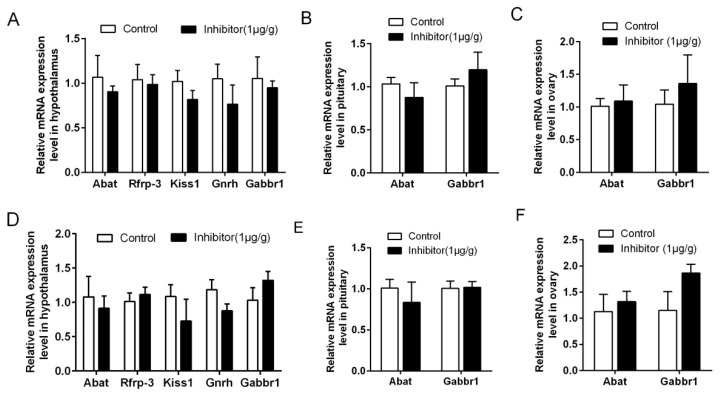
The expression of reproduction-related genes of 25d and adult rats after injected with GABA-T inhibitor for 4h. (**A**) *Abat* mRNA and reproduction-related genes levels in hypothalamus of 25d rats. (**B**) *Abat* and *Gabbr1* mRNA levels in pituitary of 25d rats. (**C**) *Abat* and *Gabbr1* mRNA levels in ovary of 25d rats. (**D**) Expression of *Abat* mRNA and reproduction-related genes in hypothalamus of adult rats. (**E**) Expression of *Abat* and *Gabbr1* mRNA in pituitary of adult rats. (**F**) Expression of *Abat* and *Gabbr1* mRNA in ovary of adult rats. Adult rat age is 64.58 ± 0.52 d. Vigabatrin was used as a GABA-T inhibitor.

In order to verify the short-term effect of the GABA-T inhibitor on reproductive genes in rats, we tested the expression of reproduction-related genes by RT-qPCR. In the rats at 25 d, the expression of hypothalamic *Abat*, *Rfrp-3*, *Kiss1*, *Gnrh* and *Gabbr1* mRNAs (Figure 5A), pituitary (Figure 5B) and ovarian (Figure 5C) *Abat* and *Gabbr1* mRNAs were not significantly different between the inhibitor group and control group (*p* > 0.05). In the adult rats, the expression levels of hypothalamic *Abat*, *Kiss1* and *Gnrh* mRNAs were decreased (Figure 5D), but *Rfrp-3* and *Gabbr1* mRNA showed no significant difference (*p* > 0.05). *Abat* and *Gabbr1* mRNA levels had no difference between the pituitary (Figure 5E) and ovary (Figure 5F) (*p* > 0.05). 

#### 3.5.2. Concentration of serum hormone after 4 h with GABT-T inhibitor injection

**Figure 6:**
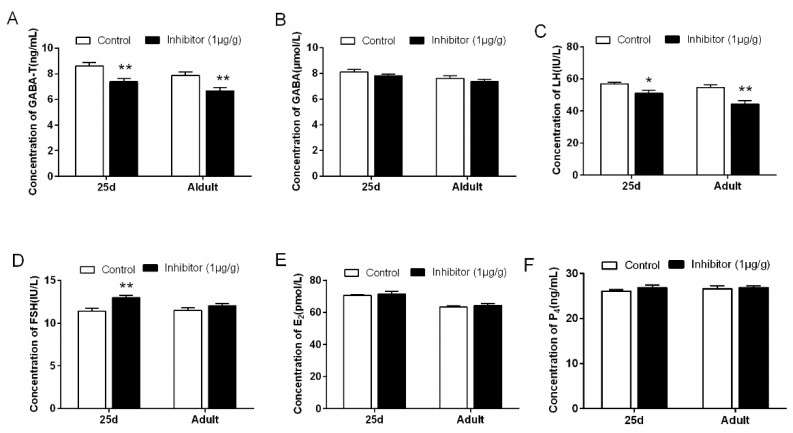
The level of serum reproductive hormone in 25d and adult rats after injected with GABA-T inhibitor for 4h. (**A**) The content of GABA-T in rat serum at 4h. (**B**) The content of GABA in rat serum at 4h. (**C**) The content of luteinizing hormone (LH) in rat serum at 4h. (**D**) The content of follicle-stimulating hormone (FSH) in rat serum at 4h. (**E**) The content of E2 in rat serum at 4h. (**F**) The content of P4 in rat serum at 4h. Adult rat age is 64.58 ± 0.52 d. ** indicates a very significant difference (*p* < 0.01); * indicates a significant difference (*p* < 0.05). Vigabatrin was used as a GABA-T inhibitor.

The serum reproductive hormone levels in 25 d and adult rats injected with 1 μg/g vigabatrin after 4 h are shown in Figure 6. We found that the serum GABA-T (*p* < 0.01) and LH (*p* < 0.05) concentrations (Figure 6A,C) in 25 d and adult rats were significantly lower in the inhibitor group than the control group, but FSH concentration (*p* < 0.01) (Figure 6D) was increased in rats at 25 d. The levels of P_4_ (Figure 6F), GABA (Figure 6B) and E_2_ (Figure 6E) in the animals at 25 d and adult were not significantly different between the two groups (*p* > 0.05).

## 4. Discussion

Our study showed that GABA-T was distributed in the reproductive axis and the expression of *Abat* mRNA in the reproductive axis changed with developmental stage in female rats. Additionally, GABA-T regulated the expression of reproduction-related genes in hypothalamus cells and affected the secretion of reproductive hormones in rats. GABA-T could also alter ovarian morphology and cause estrous cycle disorders, which could lead to early puberty and improved lactation performance.

The hypothalamus is an important organ of central nervous regulation, which can maintain normal metabolism and regulate the reproductive activities of animals. Our study suggested that GABA-T was mainly distributed in the hypothalamic ARC, PVN and PeN, similar to the distribution of GABA in the hypothalamus [11]. GABA is a major inhibitory neurotransmitter in animals, and can inhibit the synthesis of GnRH and regulate HPGA [11,37]. The neurons of Kiss1 are mainly distributed in the anterior ventral ventricular nucleus (AVPV) and ARC of the hypothalamus [38] and ARC is a key site for kisspeptin to regulate the release of GnRH pulse [37,39,40]. Kisspeptin and GABA can provide an excitatory input to GnRH neurons when co-expressed by RP3V neurons [41]. ARC, PVN and PeN are closely related to the secretion of GnRH, while GnRH is closely related to reproduction [42]. Previous studies have shown that GnRH secretion in rats changes periodically and increases in a pulsed manner before the initiation of prepuberty and decreases after the onset of puberty. GnRH thus regulates the initiation of puberty [43], and low GnRH in adults caused infertility in mice [44]. 

In our results, the expression of *Abat* mRNA showed an increasing trend from 28 d to adulthood, which is consistent with the increasing trend of GnRH and *Kiss1* levels before prepuberty to puberty [43,45], indicating that *Abat* is closely related to *Gnrh* and *Kiss1*. Studies in moles showed that *Kiss1* and *Gnrh* mRNA can be expressed in the hypothalamus and the decrease in *Gnrh* mRNA levels in adults can lead to reproductive disorders [38,46], showing that the increase in *Abat* in adults is related to the increase in GnRH during adulthood. These results showed that GABA-T regulates the periodic release of GnRH by regulating GABA in ARC, PVN and PeN or kisspeptin in ARC, thereby regulating puberty and reproduction.

The pituitary can be divided into the neurohypophysis and adenohypophysis. Athe aenohypophysis is the regulating hub of the endocrine system, which mainly receives the double regulation of the hypothalamus-releasing hormone and target adenohormone, thus affecting reproductive development [47]. Our study showed that GABA-T was mainly distributed in the adenohypophysis and *Abat* mRNA expression gradually decreased from infancy to 28 d, increased during the puberty stage, and decreased again in adulthood. Previous studies have shown that GABA can inhibit the secretion of LH and PRL through the dopamine system and the addition of GABA-T inhibitors in vitro and in vivo results in a decrease in PRL release from the pituitary [15,19,48,49]. PRL regulates the reproductive axis by acting on hypothalamic neurons expressing the *Kiss1* gene [50,51], indicating that GABA-T regulates the release of pituitary hormones (LH and PRL) via the GABAergic system, thus affecting the reproductive axis.

Our results showed that GABA-T was expressed in the ovaries at different developmental stages, and the *Abat* mRNA level was the highest in animals at 21 d, and the lowest in puberty and adulthood. GABA concentrations in peripheral organs are higher than in the central nervous system after injection of GABA-T inhibitors [52]. GABA inhibits the secretion of ovarian P_4_ and E_2_ by GABAA and GABAC receptors, which suggested that GABA-T regulates ovarian development by regulating GABA [53]. In the female rat reproductive axis, GABA-T may affect reproductive function through the GABAergic or Kiss1-GPR54 systems. Indeed, some studies have shown that kisspeptin signal can be detected in the follicles, corpus luteum and interstitial cells of adult rats [54]. 

Neurons are the ultimate target for regulators (neurotransmitters, neuropeptides, and peripheral hormones) of hypothalamic-pituitary-gonadal (HPG) axis release [8]. RFRP-3 is a mammalian ortholog of Gonadotropin-inhibitory hormone (GnIH), and can be expressed in hypothalamus ARC, PVN, terminal striate bed nucleus (BNST), preoptic medial area (MPOA), median hump (ME) and medial dorsal nucleus (DMH) [55], which inhibits GnRH and Kisspeptin neurons. The *Abat* gene encodes GABA-T, *Gabbr1* encodes the B receptor that forms GABA, *Rfrp-3* encodes RFRP-3, and *Gnrh* and *Kiss1* encode GnRH and kisspeptin, respectively. Our results showed that the levels of *Abat* mRNA, *Rfrp-3* and *Gabbr1* decreased, but the expression of *Kiss1* and *Gnrh* mRNA significantly increased after the addition of GABA-T inhibitors to hypothalamic neurons, consistent with the enhancement of GnRH neuronal activity and promotion of GnRH by GABA at the cellular level [56]. In this study, the expression of *Gabbr1* mRNA was decreased at the cellular level, indicating that GABA-T may not affect GnRH through the GABAB receptor. 

In addition, the levels of *Abat* mRNA, *Kiss1* and *Gnrh* mRNA were reduced, while the *Rfrp-3* and *Gabbr1* mRNA expression were increased in the rat at VO after GABA-T inhibitor injection into lateral ventricle. The results were contrary to the expression of reproductive genes in vitro, but supported the inhibition of GnRH release by GABA in monkeys and adult rats, and were contrary to the activation of GnRH neurons by GABA in 7 d rats and adult female sea lamprey, suggesting that in environment in vitro and vivo, species and age may influence the regulation of GnRH neurons by GABA-T [19,57,58]. However, there was no significant difference between the inhibitor group and the control group in the mRNA of *Abat* and *Gabbr1* in the pituitary and ovaries of rats during VO, indicating that GABA-T inhibitors only had a significant effect at the hypothalamus level and had a weak effect on the pituitary and ovary. Therefore, RFRP-3, kisspeptin and GABA are related to the secretion of GnRH, and GnRH is a core substance in the regulation of animal reproduction, indicating that GABA-T mainly regulates the expression of RFRP-3, kisspeptin, GnRH and GABA in the hypothalamus and neurons of female rats. 

The initiation of puberty is a complex, organized, multi-level regulated biological process, which is controlled by the reproductive neuroendocrine system and affected by environmental and genetic factors. The upstream neuropeptides that control and regulate GnRH secretion include kisseptin, neuropeptide Y (NPY), leptin (leptin), substance P (SP), neurokinin B (NKB), and RFRP-3 [59]. After the injection of GABA-T inhibitor into the hypothalamus of rats, the levels of *Rfrp-3* and *Gabbr1* mRNA in the hypothalamus were increased and *Kiss1* and *Gnrh* mRNA expression were decreased, and the VO time at puberty was significantly delayed. This was consistent with the fact that intracerebroventricular injection of RFRP-3 can significantly delay VO time and effectively inhibit the expression of *Kiss1* mRNA in female rats [59]. In addition, the serum GABA-T, LH and P_4_ levels in the inhibitor group were significantly reduced during VO. Previous studies have shown that RFRP-3, GABA and GnRH can affect the secretion of reproductive hormones, specifically, RFRP-3 inhibits the secretion of LH in adult rats, mice, hamsters and sheep [9,59], GABA inhibits the secretion of LH, E_2_, P_4_ and PRL [19,53], and GnRH promotes the secretion of LH and FSH [10]. 

Unfortunately, no difference was found in the serum concentration of GABA. This result is inconsistent with the decrease in GABA concentration of brain tissue homogenate in mice fed with vigabatrin; rats bilaterally injected with 10 μg vigabatrin into the subthalamic nucleus (STN) [22,23]; and long-term oral vigabatrin in patients with epilepsy, decreasing serum GABA levels [60]. These findings suggest that vigabatrin administration method, dose, location and species may change the effect of GABA-T on serum GABA level. In our study, the ovarian index of the inhibitor group was significantly reduced, and there was a large number of secondary follicles, while the control group had more corpus luteum, which was related to the increase in ovarian weight and luteal generation by LH [61]. These hormones are closely related to the initiation of puberty and ovarian development, suggesting that GABA-T promotes LH and P_4_ expression by inhibiting RFRP-3 and GABA and activating GnRH, and thus affecting the reproductive development of rats. However, the serum reproductive hormone levels in rats after 4 h with GABA-T inhibitor treatment were similar to those of in rats during VO, indicating that GABA-T inhibitors had a rapid and long-lasting effect on reproduction-related hormones. 

The estrous cycle in rats is composed of metestrus, estrus, proestrus and diestrus [62]. Generally, females are more receptive to male mating during estrus, and changes in the estrous cycle will affect the mating time and conception rate of animals [62,63]. In the present study, the estrous cycle of rats after GABA-T inhibitor injection was disturbed and the estrous was in the interestrus period for a long time, which was related to reduced LH levels in serum hormone. LH is one of the major hormones that regulate ovulation and maintain pregnancy and can affect the development of follicles during the luteal phase [64,65]. Previous studies have shown that LH protein and *LHR* mRNA have different expression patterns and expression levels in the estrous cycle of adult dogs, which can affect the estrous cycle of adult dogs [36]. Therefore, the decrease in GABA-T may lead to the change in LH in the serum, and thus change the estrous cycle of rats. 

Reproductive performance includes childbearing performance and lactation performance, where childbearing performance includes pregnancy rate and litter size. The weight of newborn mice in the inhibitor group was significantly lower than that in the control group, indicating that the lactation performance and milk production of female rats in the inhibitor group were decreased, which supported that GABA-T promoted PRL secretion [19]. When GABA-T was inhibited, the GABA content increased, leading to decreased PRL secretion and weight loss in newborn mice. However, treatment with GABA-T inhibitor had no effect on the reproductive performance of rats, indicating that GABA-T could promote the onset of female puberty and increase the weight of the offspring without affecting the reproductive performance. Our results suggested that GABA-T was essential in regulating the breeding time and the growth and development of the offspring.

## 5. Conclusions

In conclusion, GABA-T was expressed in the hypothalamus, pituitary and ovary of female rats at different developmental stages and the level of *Abat* mRNA in the reproductive axis changed with the development process. After the inhibition of GABA-T, the initiation of puberty was delayed, the estrous cycle was disordered, and the breastfeeding performance was reduced. In addition, the transcriptional levels of *Rfrp-3*, *Gabbr1*, *Kiss1* and *Gnrh* mRNA in hypothalamic cells were altered and serum LH and P_4_ concentrations were decreased. These results suggest that GABA-T has an effect on the reproduction of female rats. Using these results, the mechanism of GABA-T regulating reproduction will be further studied in our lab.

## Figures and Tables

**Table 1:** Primers Used for RT-PCR.

Gene	Forward primer, 5′–3′	Reverse primer, 5′–3′	Product Length, (bp)
*Abat*	GGTCATCAACATCATCAAG	TTATTCCGTATGGCTTCG	174
*Gnrh*	GCCGCTGTTGTTCTGTTGAC	CTGGGGTTCTGCCATTTGA	134
*Rfrp-3*	CCAAAGGTTTGGGAGAACAA	GGGTCATGGCATAGAGCAAT	127
*Kiss1*	TGCTGCTTCTCCTCTGTG	CCAGGCATTAACGAGTTCC	116
*Gabbr1*	CACGAAGAAGGAGGAGAAG	CAGATGGCAAGAGTCAGG	108
*Gapdh*	TCAACGGCACAGTCAAGG	CTCAGCACCAGCATCACC	113
*β-actin*	CGTGACATCAAGGAGAAG	GAAGGAAGGCTGGAAGAG	171

**Table 2:** Ovarian weight and size at vaginal opening (VO) after GABA-T inhibitor injection.

Group	Weight (mg)	Transverse Diameter (cm)	Longitudinal Diameter (cm)	Transverse Perimeter (cm)	Longitudinal Perimeter (cm)	Ovary Index
Control	20.83 ± 1.913	0.486 ± 0.014	0.326 ± 0.016	1.238 ± 0.072	0.936 ± 0.017	0.216 ± 0.163
Inhibitor (1μg/g)	20.23 ± 1.356	0.504 ± 0.024	0.317 ± 0.009	1.231 ± 0.055	0.913 ± 0.022	0.172 ± 0.011 *

All data are shown as mean ± SEM, * indicates a significant difference (*p* < 0.05). Vigabatrin was used as a GABA-T inhibitor.

**Table 3:** Effects of GABA-T inhibitor treatment on distribution of time of conception in rats during 21 d mating period.

Time (d)	Number of Pregnant Rat and Precent (%)	
	Control (n = 10)	Inhibitor (n = 10)
1	1 (10%)	3 (30%)
2	3 (30%)	2 (20%)
3	1 (10%)	1 (10%)
4	4 (40%)	4 (40%)
5~12	0 (0%)	0 (0%)
13	1 (10%)	0 (0%)
14~21	0 (0%)	0 (0%)
Total	10 (100%)	10 (100%)

Vigabatrin was used as a GABA-T inhibitor.

**Table 4:** Effects of GABA-T inhibitor treatment on pregnancy in female rats.

Period (d)	Number of Pregnant Rat and Precent (%)	
	Control (n = 10)	Inhibitor (n = 10)
22	4 (40%)	4 (40%)
23	6 (60%)	6 (60%)
Total	10 (100%)	10 (100%)

Vigabatrin was used as a GABA-T inhibitor.

**Table 5:** Effects of GABA-T inhibitor treatment on litter size in female rats.

Age (d)	Control (n = 10)	Inhibitor (n = 10)
0	17.30 ± 2.71	15.50 ± 2.50
4	16.30 ± 2.71	15.10 ± 2.51
7	8.00 ± 0.00	8.00 ± 0.00
14	7.70 ± 0.15	8.00 ± 0.00
21	7.70 ± 0.15	8.00 ± 0.00

All data are shown as mean ± SEM. Vigabatrin was used as a GABA-T inhibitor.

## Supplementary Materials

The following are available online at https://www.mdpi.com/2076-2615/10/4/567/s1, Figure S1 Prepuberty (A) and puberty (B) characteristics of the vulva. The red arrow indicates the rat’s vulva, Figure S2 Vaginal smear in rat estrous cycle. DI:diestrus; P:preoestrus; E:estrus; M:metaoestrus.
