# Supplementary material for: Effect of GABA-T on Reproductive Function in Female Rats
Source: Animals (Basel). 2020 Mar 27;10(4):567. doi: 10.3390/ani10040567 (PMC7222393; doi:10.3390/ani10040567)
Supplement: Supplementary file 1 [file animals-10-00567-s001.pdf]

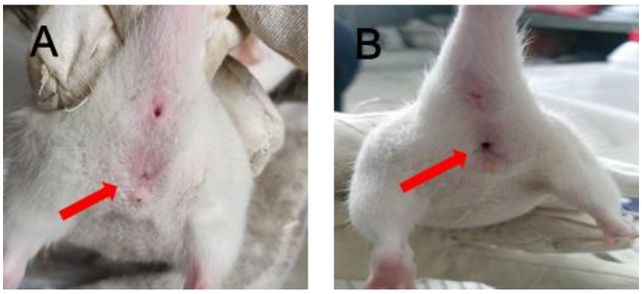

Supplementary Figure 1 Prepuberty (A) and puberty (B) characteristics of the vulva. The red arrow indicates the rat's vulva.

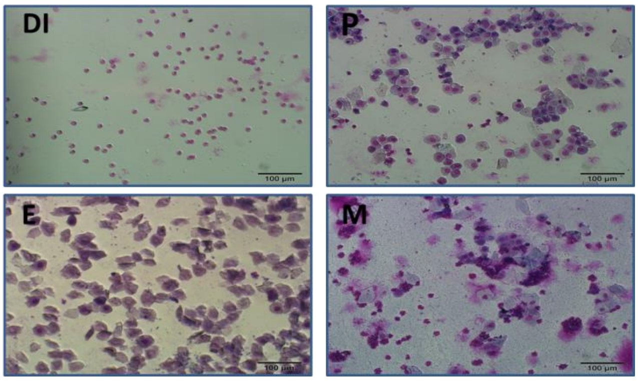

Supplementary Figure 2 Vaginal smear in rat estrous cycle. DI:diestrus; P:preoestrus; E:estrus; M:metaoestrus.
